# Effects of the biodegradable film-based tray-free seedling system on seedling quality and ecological adaptability in cold-region rice

**DOI:** 10.1371/journal.pone.0361056

**Published:** 2026-10-27

**Authors:** Zhong Yi Sun, Qing Hua Kang, Hong Da Wu, Shi Wei Zhou, Dan Dan Yao, Xi Xia Song, Chun Xu Leng, Ping Yan, Hong Tao Wu, Hai Ying Liu, Hong Ru Gao, Bei Ping Zhao, Yu Fu Wang, Zhen Hua Xu

**Affiliations:** 1 Institute of Biotechnology, Heilongjiang Academy of Agricultural Sciences, Harbin, China; 2 Wuchang Experimental Station, Northeast Center, National Technology Innovation Center for Saline-Alkali Tolerant Rice, Harbin, China; 3 Institute of Industrial Crops, Heilongjiang Academy of Agricultural Sciences, Harbin, China; 4 Institute of Bast Fiber Crops, Chinese Academy of Agricultural Sciences, Changsha, China; Institut National de la Recherche Agronomique, MOROCCO

## Abstract

To reduce labor costs, mitigate plastic pollution, and enhance disease control in cold-region rice seedling nurseries, this study developed a biodegradable film-based seedling system.From 2022 to 2024, large-scale comparative field experiments were conducted over three consecutive years in seven major rice-producing cities and counties in Heilongjiang Province, including Tailai, Tonghe, and Fangzheng, to systematically analyze the effect of this technique on seedling morphology, biomass, vigor, and other key indicators, as well as its ecological and inter-annual adaptability. The results showed that, compared with traditional mechanical transplanting tray seedling raising (CK), the biodegradable film-based treatment (T1) increased seedling height, root length, stem base width,dry weight per hundred seedlings, and the classic strong seedling index (SRI) by an average of 6.82%, 8.97%, 9.52%, 10.26%, and 12.41%, respectively, while maintaining a stable root-shoot ratio and water metabolism.No bacterial wilt or seedling blight was observed throughout the experimental period, and no white plastic residue was detected.The total costof seedling raising was reduced by 15%–20%. Regionally, the technique performed best in high-biomass zones (e.g., Tonghe, Mishan) and balanced robust seedling zones (e.g., Suibin, Qing’an), while also showing positive effects in stress-prone regions. This technique demonstrates potential for improving seedling quality while reducing costs and plastic residue.Across 21 site-year combinations spanning seven major rice-producing regions, the system demonstrated consistent positive effects (T1/CK ratios >1.02) with the highest advantages observed in high-biomass regions (Tonghe and Mishan), confirming broad ecological adaptability.

## 1. Introduction

As China’s largest commercial grain base, the rice planting area and yield in Heilongjiang Province are of strategic significance for ensuring national food security [1,2]. Seedling raising is a critical step in cold-region rice cultivation. However, traditional seedling raising models have long faced multiple drawbacks, making it difficult to meet current production development needs [3]. In terms of labor, this model requires high labor input and technical expertise, conflicting with the current trend of an aging and labor-shortage agricultural labor force, directly increasing production costs [4]. At the production and management level, with the acceleration of intensive land transfer, new agricultural entities such as large-scale growers and family farms have become the main production force, and traditional seedling raising models are inadequate in terms of efficiency and scale [5].Interms of ecological and environmental protection, traditional mechanical transplanting tray seedling raising heavily relies on non-degradable plastic trays, leading to severe agricultural white pollution [6,7]. Furthermore, the special ecological conditions in cold regions further amplify the shortcomings of traditional models: low temperatures in early spring and slow soil warming not only increase the risk of chemical damage in the seedbed but also lead to frequent seedling diseases such as bacterial wilt and seedling blight [8], ultimately resulting in uneven seedling quality and a high rate of missing plants during mechanical transplanting, which severely affects rice yield formation. Therefore, developing a new seedling-raising technology that balances simplified operation, large-scale production, and environmental protection has become an urgent task for promoting the high-quality development of the rice industry in cold regions [9,10].

We hypothesized that the biodegradable seedling film would optimize the rhizosphere microenvironment by improving heat preservation, moisture conservation, and aeration, thereby alleviating root growth inhibition caused by early spring low temperatures and promoting coordinated root-shoot development in cold-region rice nurseries.

## 2. Materials and methods

### 2.1. Experimental conditions

The experiments were conducted from 2022 to 2024 in seven major rice-producing cities and counties in Heilongjiang Province: Tailai, Tonghe, Fangzheng, Qing’an, Mishan, Suibin, and Fuyuan. Each experimental site had a solid foundation for rice production. The experimental fields were open with flatterrain, had uniform soil fertility,and had convenient water resources for irrigation and drainage, and were capable of representing the typical climate and soil conditions of the local area. The geographical, climatic, and soil physicochemical properties of each experimental site are shown in Table 1. Geographical coordinates were determined using GPS, climate data were the average values during the experimental period (2022–2024), and soil indicators were determined using conventional methods [11].

**Table 1:** Basic information and soil physicochemical properties of the experimental sites.

Experimental Site	Latitude and Longitude	≥10°C Effective Accumulated Temperature (°C)	Frost-Free Period (d)	Annual Precipitation (mm)	Soil Type	Organic Matter Content (g/kg)	pH Value	Alkali-Hydrolyzable Nitrogen (mg/kg)	Available Phosphorus (mg/kg)	Available Potassium (mg/kg)
Tailai County	N46°33′-47°00′, E123°34′-124°56′	2850 ± 52	142 ± 7	400 ± 35	Meadow Chernozem	28.6 ± 2.3	7.2 ± 0.3	125.4 ± 8.6	23.8 ± 3.1	186.5 ± 12.4
Tonghe County	N45°53′-46°40′, E128°09′-129°25′	2600 ± 48	135 ± 6	650 ± 42	Dark Brown Soil	35.2 ± 3.1	6.8 ± 0.2	142.7 ± 9.3	28.5 ± 2.7	203.6 ± 10.8
Fangzheng County	N45°32′-46°09′, E128°13′-129°04′	2550 ± 45	130 ± 5	620 ± 38	Black Soil	32.8 ± 2.7	6.9 ± 0.2	138.2 ± 7.9	25.3 ± 2.4	195.8 ± 11.2
Qing’an County	N46°30′-47°35′, E126°14′-127°45′	2700 ± 50	138 ± 6	580 ± 40	Chernozem	30.5 ± 2.5	7.0 ± 0.3	131.6 ± 8.1	24.6 ± 2.9	190.3 ± 13.1
Mishan City	N45°14′-46°37′, E131°14′-133°08′	2650 ± 46	132 ± 5	550 ± 36	Meadow Soil	33.7 ± 2.9	6.7 ± 0.2	145.3 ± 9.6	29.1 ± 3.2	210.7 ± 12.6
Suibin County	N47°11′-47°45′, E131°8′-132°31′	2500 ± 42	128 ± 5	500 ± 32	Bog Soil	36.4 ± 3.2	6.6 ± 0.2	150.2 ± 10.4	30.4 ± 2.8	215.4 ± 11.9
Fuyuan City	N47°25′-48°27′, E133°40′-135°05′	2350 ± 38	120 ± 4	480 ± 30	Albic Soil	26.3 ± 2.1	6.5 ± 0.2	118.5 ± 7.5	22.7 ± 2.3	178.6 ± 10.5

Note: 1. Climate data were obtained from the meteorological bureaus of the cities/counties where the experimental sites are located; 2. Soil organic matter was determined by the potassium dichromate volumetric method, pH was determined by the potentiometric method (soil:water ratio 1:2.5), alkali-hydrolyzable nitrogen was determined by the diffusion method, available phosphorus was determined by the molybdenum-antimony anti-colorimetric method, and available potassium was determined by the flame photometric method; 3. Climate data are presented as mean ± standard deviation of the experimental period (2022–2024).

### 2.2. Varieties used in the experiment

The rice varieties used at each experimental site were nationally or provincially approved main cultivars recognized by the Provincial Agricultural Extension Center, ensuring suitability for local climate and cultivation conditions [12]. Rice varieties used at each experimental site: Tailai County (Longyang 16, Longdao 124, Zhongkefa 5), Tonghe County (Shansidao 8, Tiannong 17, Suijing 9), Fangzheng County (Fangxiangdao 2, Tianhe 1), Qing’an County (Longjing 3010, Zaodaoxiang, Chuxiangjing 1), Mishan City (Suijing 18, Longjing 31, Longdao 1), Suibin County (Longjing 31, Longjing 1718, Lianhui 6730), Fuyuan City (Longjing 31, Lianhui 13, Longjing 3049).

### 2.3. Test materials

The core material was the naturally biodegradable environmentally friendly seedling film developed by the Institute of Biotechnology, Heilongjiang Academy of Agricultural Sciences [13]. This film is made from natural plant fibers, supplemented with compound amino acids and trace elements, with a thickness of0.8–1.0 mmand a width of 1.5 m. Thesupporting materials included a biodegradable perforated isolation film (thickness: 0.01 mm, width: 3 m,hole diameter: 0.5 cm,hole spacing: 5 cm × 5 cm) and special nutrient soil for seedling raising (organic matter content ≥ 30%). The control group (CK) used traditional plastic trays (58 cm × 28 cm,holes diameter: 0.3 cm,hole spacing: 5 cm × 5 cm).

### 2.4. Experimental methods

A large-area comparative method was adopted with each treatment applied in a single large plot (≥150 m²) without field replication within each site; however, the experimental design relied on multi-site and multi-year ecological replicates (21 site-year combinations) to assess treatment effects. This approach has been widely employed for evaluating the broad adaptability of new agricultural technologies across varying environmental conditions [14].

Five sampling points were randomly selected per treatment at each site, yielding 105 replicate samples per treatment (5 sampling points × 21 site-year combinations), with 20 seedlings measured per sampling point.

**Treatment (T1):** Biodegradable film-based tray-free seedling system for cold regions. The technical process of this technique is as follows: leveling the seedbed → laying the isolation film → laying the biodegradable seedling film (pressing the edges) → spreading 2 cm of nutrient base soil → watering thoroughly → sowing (sowing rate consistent with local convention, 600 g/m² dry seed) → covering with 0.5 cm of topsoil → covering with a plastic film → conventional greenhouse management [13,15].

**Control (CK):** Traditional tray seedling raising. The technical processof this technique is as follows:leveling the seedbed → laying gauze fabric or fine sand → placing trays → spreading 2 cm of nutrient base soil → watering thoroughly → sowing (sowing rate consistent with local convention, 600 g/m² dry seed) → covering with 0.5 cm of topsoil → covering with a plastic film → conventional greenhouse management.

Within the same experimental greenhouse, the variety was kept consistent, and management measures such as temperature, humidity, ventilation, and disease control were uniformly executed by trained personnel to ensure consistent conditions. The area for each treatment was ≥ 150 m², and the seedling age was controlled at 25–38 days (adjusted according to variety and region).

### 2.5. Measurement indicators and methods

When the seedlings reached the optimal age for mechanical transplanting, five sampling points were randomly selected for each treatment (T1) and control (CK) at each experimental site. At each sampling point, twenty seedlings (n = 20) were randomly selected to measure the following indicators:

**Morphological indicators:** Plant height (from soil surface to the tip of the highest leaf, cm), root length (from the base to the tip of the longest root, cm), stem base width (diameter at 1 cm from the base, cm), tiller number (number of effective tillers per seedling), leaf age (number of fully expanded leaves).

**Biomass indicators:** Fresh weight of above-ground and below-ground parts per 100 seedlings (g). After killing at 105°C for 30 minutes and drying at 80°C to constant weight, the dry weight of above-ground and below-ground parts per 100 seedlings (g) was measured. The root-shoot ratio offresh weight: below-ground fresh weight/above-ground fresh weight, the root-shoot ratio of dry weight: below-ground dry weight/above-ground dry weight, dry-fresh ratio: dry weight/fresh weight.

**Vigor indicators:** The strong seedling index(SRI) was calculated using the classic formula: SRI = (stem base width/ plant height) × above-ground dry weight [16]. Stockiness (mg/cm) was calculated as above-ground dry weight (mg)/ plant height (cm).Plumpness (%) was calculated as (total dry weight per 100 seedlings (mg)/ total fresh weight per 100 seedlings (mg)) × 100%.Above-ground and below-ground strong seedling indices were not used in the final analysis; only the classic SRI was adopted for consistency.

**Disease incidence:** The incidence rates (%) of bacterial wilt and seedling blightwere investigated.

### 2.6. Data statistical analysis

Excel 2021 was used for data organization and mean calculation. SPSS 26.0 was used for significance testing (independent samples t-test), with *P* < 0.05 considered statistically significant and *P* < 0.01 considered highly significant. Origin 2023 was used for chart plotting. Data were presented asmeans ± standard deviations. Data from 21 site × year combinations (7 sites × 3 years) were combined to analyze the overall effect of the technique as well as regional and interannual differences.

## 3. Results and discussion

### 3.1. Effects of biodegradable film-based tray-free seedling system on seedling morphological indicators

According to the measurement results from the 7 experimental sites from 2022 to 2024 (Table 2), the biodegradable film-based tray-free system (T1) significantly improved the morphological indicators of seedlings compared to traditional seedling raising (CK). The average plant height of T1 seedlings was 13.86 cm, representing an increase of 6.82% compared to CK (*P* < 0.05); the average root length was 3.82 cm, an increase of 8.97% (*P* < 0.05); the average stem base width was 0.25 cm, an increase of 9.52%, and the difference was highly significant (*P* < 0.01); the average leaf age was 3.42 leaves, which increased slightly by (2.09%), but the difference was not significant (*P* > 0.05). Notably, the T1 treatment promoted early tillering during the seedling stage, with an average tiller number of 0.18 per seedling (range: 0.16–0.2), while CK had no tillers, and the difference was highly significant (*P* < 0.01).

**Table 2:** Statistical analysis of seedling morphological indicators for tray-free and traditional seedling raising methods in cold-region rice (2022–2024).

Indicator	Treatment Group (T1) (Mean ± SD)	CK (Mean ± SD)	Average Increase (%)	*P*-value
Plant Height (cm)	13.86 ± 1.52	12.97 ± 1.48	6.82	<0.05
Root Length (cm)	3.82 ± 0.95	3.50 ± 0.89	8.97	<0.05
Stem Base Width (cm)	0.25 ± 0.07	0.23 ± 0.06	9.52	<0.01
Tiller Number (per plant)	0.18 ± 0.32	0.00 ± 0.00	**—**	**—**
Leaf Age (leaf)	3.42 ± 0.31	3.35 ± 0.29	2.09	>0.05

Note: Data are averaged across varieties from 7 experimental sites during 2022–2024.An em dash (—) indicates that no corresponding statistical value was calculated.No tillers were observed in CK.

From an agronomic standpoint, the synergistic optimization of these morphological indicators collectively constructs an ideal physiological architecture for high-yielding and robust seedlings. The highly significant increase in stem base width directly enhances seedling sturdiness and lodging resistance potential, laying a solid foundation for subsequent transport and support [17]. The significant increase in root length implies that seedlings develop a more powerful absorption and anchoring system, facilitating rapid greening after transplanting and enhancing drought resistance [18]. The moderate increase in plant height, coupled with increased stem base width, is beneficial for light competition within the canopy. The appearance of tillers during the seedling stage is among the most direct indicators of yield increase potential; these early tillers are expected to directly translate into effective panicles during the main field stage.

By cultivating high-quality robust seedlings characterized by thick stem bases, long roots, and tillering capacity, the T1 treatment exhibits characteristics of optimizing core indicators while stabilizing basic functions. Such robust seedlings not only possess better stress resistance and survival rates but also promote early and rapid growth after transplanting, thereby prolonging the effective tillering period and laying a solid foundation for increasing effective panicle number per unit area and achieving stable and high yields. This technique precisely enhances traits directly related to yield and stress resistance, making it suitable for large-scale planting needs and serving as an effective alternative to traditional tray seedling raising techniques.

### 3.2. Effects of biodegradable film-based tray-free seedling system on seedling vigor indicators

Based on the measurement data from the 7 experimental sites from 2022 to 2024, the seedling vigor indicators of the treatment (T1) and control (CK) were analyzed. The results indicated that the biodegradable film-based tray-free system could significantly optimize the comprehensive quality of seedlings, and its effects showed regular differences across different ecological regiontypes. The specific statistical results are shown in Table 3.

**Table 3:** Comparison of seedling vigor indicators between tray-free and traditional seedling raising methods in different ecological zones.

Ecological Zone	Treatment	Strong Seedling Index	Stockiness (mg·cm^-1^)	Plumpness (%)
High Biomass Region (Tonghe, Mishan)	T1	0.097 ± 0.023	2.31 ± 0.45	19.8 ± 3.2
	CK	0.087 ± 0.020	2.09 ± 0.41	19.5 ± 3.5
Balanced Robust Seedling Region (Qing’an, Suibin)	T1	0.058 ± 0.015*	1.85 ± 0.31*	21.5 ± 2.8
	CK	0.049 ± 0.013	1.68 ± 0.28	21.0 ± 3.1
Compact Stress-Tolerant Region (Fangzheng, Fuyuan)	T1	0.042 ± 0.011	1.33 ± 0.25	23.2 ± 4.1
	CK	0.036 ± 0.009	1.21 ± 0.22	22.8 ± 4.5
Unique Tillering Region (Tailai)	T1	0.040 ± 0.003	1.05 ± 0.30	22.8 ± 1.5
	CK	0.037 ± 0.003	1.03 ± 0.28	22.9 ± 1.7

Note**:** Values are presented as means ± standard deviations (SD). T1: biodegradable film-based tray-free seedling system; CK: conventional tray seedling raising control. Strong seedling index is dimensionless; stockiness is expressed as dry weight per unit plant height; plumpness is the dry matter percentage per seedling. An independent samples t-test was conducted for comparison between T1 and CK within each ecological zone. indicates a significant difference at *P* < 0.05. No significant differences were observed for other indicators (*P* > 0.05).

Compared with CK, the T1 treatment enhanced the core comprehensive vigor of seedlings in different ecological regiontypes. Among them, the strong seedling index showed a significant increase (*P* < 0.05) in the high biomass region and the balanced robust seedling region, with average increases of 11.5% and 18.4%, respectively. Looking at sub-indicators, the above-ground strong seedling index and below-ground strong seedling index also showed a consistent increasing trend in most ecological regiontypes. The seedling stockiness increased significantly (*P* < 0.05) in the high biomass region and the balanced robust seedling region, indicating that dry matter accumulation per unit plant height was more substantial, which is conducive to cultivating stocky and lodging-resistant seedlings [19]. Additionally, there were no significant differences in seedling plumpness between T1 and CK in any ecological regiontypes, indicating that while promoting growth, the technique ensured the compactness of seedling tissue without affecting its stress resistance.

By promoting coordinated root-shoot growth and optimizing morphological architecture, the treatment (T1) effectively cultivated robust seedlings with thick stems, developed roots, and substantial material accumulation, laying a good foundation for high and stable yields of cold-region rice after transplanting.

### 3.3. Effects of biodegradable film-based tray-free seedling system on seedling biomass indicators

Biomass is a core indicator reflecting the seedling’s ability to accumulate nutrients. Table 4 shows that the fresh weight and dry weight per 100 seedlings of the treatment (T1) were significantly higher than those of CK. Biomass analysis showed that the average above-ground fresh weight per 100 seedlings in the treatment group was 11.89g, an increase of 7.89% compared to CK; the average below-ground fresh weight was 8.76g, an increase of 9.77%; the average above-ground dry weight was 2.15g, an increase of 10.26%; the average below-ground dry weight was 1.72g, an increase of 10.25%, indicating that the treatment (T1) could simultaneously promote the accumulation of above-ground and below-ground biomass, with a slightly better promotion effect on the below-ground part compared to the above-ground part.

**Table 4:** Summary and analysis of seedling biomass indicators for tray-free and traditional seedling raising methods in cold-region rice (2022–2024).

Indicator	Treatment Group (T1) (Mean ± SD)	CK (Mean ± SD)	Average Increase (%)	*P*-value
Above-ground Fresh Weight per 100 seedlings (g)	11.89 ± 4.21	11.02 ± 3.98	7.89	<0.05
Below-ground Fresh Weight per 100 seedlings (g)	8.76 ± 2.85	7.98 ± 2.67	9.77	<0.05
Above-ground Dry Weight per 100 seedlings (g)	2.15 ± 0.89	1.95 ± 0.82	10.26	<0.01
Below-ground Dry Weight per 100 seedlings (g)	1.72 ± 0.65	1.56 ± 0.61	10.25	<0.01
Root-Shoot Ratio	0.80 ± 0.15	0.81 ± 0.14	−0.12	>0.05
Above-ground Dry-Fresh Ratio	0.18 ± 0.04	0.18 ± 0.03	0	>0.05
Below-ground Dry-Fresh Ratio	0.19 ± 0.05	0.20 ± 0.04	−0.01	>0.05

Note: Data are presented as means ± standard deviations.*P*-valuesare fromindependent samples t-tests comparing T1 and CK.

The average root-shoot ratio of the treatment (T1) was 0.80, and that of CK was 0.81, with no significant difference; the dry-fresh ratios for above-ground and below-ground parts were 0.18 and 0.19, respectively, while those of CK were 0.18 and 0.20, respectively, with no statistically significant differences. This indicates that while promoting growth, the treatment (T1) can maintain the root-shoot balance and water metabolism stability of the seedlings [20].

It is hypothesized thatthe technique centered on the biodegradable seedling film mayoptimize the rhizosphere microenvironment by improving the heat preservation, moisture conservation, and aeration of the rhizosphere soil [21]. This helps alleviate the inhibition of root growth by early spring low temperatures, promotes root elongation (root length increased by 8.97%), thereby prioritizing below-ground biomass accumulation. The developed root system further enhances the absorption capacity of water and nutrients, thus forming a positive feedback loop of “root development – nutrient absorption – above-ground growth” [22], laying a physiological foundation for cultivating robust seedlings in cold regions. Secondly, the simultaneous and highly significant accumulation of above-ground and below-ground dry matter can compensate for the photosynthetic product shortage caused by insufficient accumulated temperature during the seedling raising period in cold regions, enhancing the seedling’s material reserves [23]. Furthermore, the stable root-shoot ratio indicates that the technique promotes the construction of a reasonable plant type characterized by “strong roots and robust seedlings”, conforming to the material distribution pattern of robust seedlings in cold regions, which is conducive to rapid greening and early vigorous growth after transplanting. Finally, the maintenance of a stable dry-fresh ratio ensures appropriate cell water content and stable membrane systems in seedlings, enhancing their ability to resist adversities such as late spring cold and low-temperature, high-humidity conditions.

The regulation of biomass by the treatment (T1) is not a simple morphological increase but an overall optimization of physiological metabolism based on the ecological characteristics of cold regions, achieving the synergistic effect of **“**promoting growth, maintaining balance, and enhancing stress resistance**”**, laying a solid physiological foundation for the promotion and application of this technique in cold-region rice areas.

It should be noted that this experiment covered multiple ecological zones and different years. The absolute values of seedling biomass at each pilot site varied greatly due to local climate, variety, and cultivation management, resulting in high data variability. However, through the integrated analysis of multi-site and multi-year data, the treatment (T1) showed a stable and consistent increasing trend relative to the control (CK).The raw phenotypic data supporting all analyses are provided in S1 Dataset. The distribution of seedling morphological data is visualized in S1 Fig, and biomass variation is shown in S2 Fig. Differences in core seedling-quality traits among ecological zones are presented in S3 Fig.

### 3.4. Analysis of seedling quality differences across different years and regions

#### 3.4.1. Analysis of inter-annual seedling quality differences and their correlation with climatic conditions.

Multi-site experiments from 2022 to 2024 showed (Tables 5 and 6) that seedlings from the treatment (T1) performed better than the control (CK) in several quality indicators. Although data from each experimental site exhibited some variation due to factors including changes in varieties and fluctuations in seedling age (25–38 d), statistical analysis revealed that the differences in core seedling quality indicators (root length, dry weight per 100 seedlings, root-shoot ratio, stem base width, and the comprehensive strong seedling index) between treatment (T1) and control (CK) across the three years 2022, 2023, and 2024 were not statistically significant (*P* > 0.05). This indicates that the year factor itself had a relatively small impact on the core quality of seedlings, and the applied technique exhibited stable performance across different years.

**Table 5:** Inter-annual statistical comparison of core seedling quality parameters under tray-free rice seedling raising (T1) in cold regions (2022-2024).

Indicator	2022	2023	2024	*P*-value	Significance
Root Length (cm)	3.71 ± 0.95	3.66 ± 0.94	4.00 ± 1.31	0.782	Not Significant
Dry Weight per 100 seedlings (g)	361.6 ± 61.7	332.0 ± 80.6	424.1 ± 204.3	0.385	Not Significant
Root-Shoot Ratio (dry)	0.60 ± 0.32	0.78 ± 0.35	0.70 ± 0.20	0.476	Not Significant
Stem Base Width (cm)	0.244 ± 0.051	0.231 ± 0.053	0.266 ± 0.099	0.652	Not Significant
Strong Seedling Index	0.855 ± 0.347	0.560 ± 0.211	1.229 ± 1.270	0.211	Not Significant

Note**:** The strong seedling index wascalculated using the classic formula defined in Section 1.5.*P*-value > 0.05 indicates no significant difference between years.

**Table 6:** Inter-annual statistical comparison of core seedling quality parameters under conventional rice seedling raising (CK) in cold regions (2022-2024).

Indicator	2022	2023	2024	P-value	Significance
Root Length (cm)	3.32 ± 0.92	3.39 ± 0.95	4.00 ± 1.10	0.285	Not Significant
Dry Weight per 100 seedlings (g)	318.4 ± 66.2	278.9 ± 78.3	379.5 ± 185.7	0.424	Not Significant
Root-Shoot Ratio (dry)	0.57 ± 0.33	0.80 ± 0.36	0.70 ± 0.19	0.325	Not Significant
Stem Base Width (cm)	0.221 ± 0.056	0.207 ± 0.071	0.240 ± 0.111	0.766	Not Significant
Strong Seedling Index	0.622 ± 0.320	0.418 ± 0.169	1.036 ± 1.213	0.284	Not Significant

Note**:** The strong seedling index wascalculated using the classic formula defined in Section 1.5.No significant differences were observed for any indicator across years (*P* > 0.05).

The climatic patterns during the experimental period were distinct: the spring of 2022 experienced low temperatures and late spring cold, leading to insufficient accumulated temperature; 2023 was characterized by early drought followed by late waterlogging,with periodic low temperature and low light during the mid-to-late seedling stage; 2024 was a year with relatively coordinated light, temperature, and water conditions. However, under such variable climatic backgrounds, the core growth indicators of the seedlings did not show significant inter-annual fluctuations. For example, root length and dry weight per 100 seedlings did not show statistically significant decreases under the unfavorable climates of 2022 and 2023, and although their average values increased under the favorable climate of 2024, the inter-annual differences were also not significant. This suggests that the basic growth of seedlings, whether in the treatment or control, exhibited a certain adaptability or buffering capacity to the climatic fluctuations during the experimental period.

The strong seedling index, as a comprehensive physiological vigor indicator, also did not show statistically significant changes over the three years for either the treatment or control. It is worth noting that the three-year average of the strong seedling index for the treatment group was 0.881, higher than the control group’s 0.692, reflecting the positive effect of the applied technique in improving the comprehensive quality of seedlings,an effect that remained stable across different years. Importantly, no bacterial wilt or seedling blight occurred in any pilot site over the three years, highlighting the disease prevention and control effect of this technique achieved through isolation by the biodegradable film and optimization of water**–**air balance, an advantage that remains stable across inter-annual climate fluctuations.

Furthermore, variety selection and agronomic management are important factors influencing technique performance. The high strong seedling index value observed at the Suibin pilot site in 2024 (variety Lianhui 6730) contributed to raising the average level for that year, which may result from the synergistic effect of specific variety characteristics and local cultivation conditions, rather than being purely climate-driven. This suggests that achieving an integration of climate-technique-variety-management is key to obtaining better seedling performance.

In summary, the biodegradable film-based tray-free seedling system demonstrated stable application value under different climatic conditions across years: although it did not cause significant inter-annual fluctuations in core seedling indicators in response to climatic variations, it consistently resulted in seedling quality (e.g., strong seedling index) superior to that of traditional methods and exhibited strong stability in disease prevention and control. Future applications could further combine variety screening and agronomic management optimization to enhance the overall performance and adaptability of this technique under the variable climatic conditions of cold regions.

#### 3.4.2. Ecological differences.

This study analyzed seedling quality data from seven experimental sites (Tailai, Tonghe, Fangzheng, Qing’an, Mishan, Suibin, Fuyuan) from 2022 to 2024, to clarify the adaptability of the treatment (T1) in different ecological zones. The results showed (Table 7): No bacterial wilt or seedling blight occurred at any site, indicating good disease control in the seedling environment. However, seedling morphological indicators, biomass accumulation, seedling vigor quality, and the synergistic effect of treatment (T1) all showed significant regional differences.

**Table 7:** Statistics of three-year average values for key morphological and quality indicators in the treatment group (T1) across seven trial sites (2022–2024).

Experimental Site	Plant Height (cm)	Root Length (cm)	Stem Base Width (cm)	Tiller Number	Strong Seedling Index (VSI)	Total Dry Weight per 100 seedlings (g)	Root-Shoot Ratio (dry)	Plumpness (%)
Tailai	15.70	2.35	0.18	0.31	0.0399	3.36	1.11	22.68
Tonghe	14.80	4.57	0.30	0.00	0.0863	4.23	0.55	19.42
Fangzheng	13.40	4.00	0.20	0.00	0.0397	2.98	0.53	25.78
Qing’an	13.27	4.17	0.24	0.00	0.0554	4.57	0.75	21.37
Mishan	12.77	4.00	0.30	0.00	0.0815	3.57	0.46	17.32
Suibin	13.83	4.33	0.33	0.00	0.0847	4.79	0.73	21.60
Fuyuan	11.90	3.71	0.19	0.00	0.0518	2.47	0.42	23.69

Note: Some indicators atthe Suibin site in 2024 (e.g., strong seedling index) peaked, but the three-year average was influenced by data from the previous two years. Fangzheng adopted a 25-day short seedling age strategy.

The direction and magnitude of the synergistic effect produced by the same seedling raising technique varied significantly across different ecological zones. Among them, the effect of treatment (T1) was most comprehensive in Suibin and Qing’an, where it had the most significant impact on improving comprehensive quality indicators such as the strong seedling index and plumpness. These sites had the highest total dry weight (>4.57 g), thick stem bases, and healthy root-shoot ratios (>0.73).Tailai exhibited a distinct synergistic direction; treatment (T1) significantly increased the tiller number (0.31), but resulted in the highest root-shoot ratio (1.11) and the lowest strong seedling index (0.0399), with limited improvement in other morphological indicators. In Fangzheng and Fuyuan, treatment (T1) effectively compensated for local weak indicators such as root length and stem base width. These seedlings had the highest plumpness (>23.7%), the shortest plant height (<13.4 cm), and low total dry weight. The synergistic effect of treatment (T1) in Tonghe and Mishan was unbalanced. While maintaining high biomass, treatment (T1) had a relatively limited effect on improving their low root-shoot ratio (<0.56) and plumpness, although their stem base width (0.30 cm) and strong seedling index (>0.08) were relatively high.

In terms of comprehensive quality indices, regional differences in the strong seedling index, plumpness, and stockiness were significant, reflecting different characteristics in seeding quality: Tonghe, Suibin, and Mishan had higher strong seedling indices (>0.08) due to thick stems (stem base width 0.30**–**0.33 cm) and large biomass; Fangzheng and Fuyuan excelled in plumpness due to high dry matter content (plumpness >23.7%), indicating compact seedling structure and strong stress resistance potential; Suibin and Qing’an led in stockiness, with the largest dry weight per unit plant height, indicating the most stocky and robust seedlings [24].

Research indicates that there are significant ecological regional differences in the quality of cold-region rice seedlings, mainly influenced by geographical environment, climatic conditions, and seedling raising strategies, forming four identifiable types. Although the tray-free seedling raising technique possesses good universality in disease prevention and control, its regulatory effect on seedling growth shows significant interaction with the environment. Therefore, nosingle optimal solution applies to all regions. Promotion must be tailored to local conditions and precisely adapted: In high biomass regions like Tonghe and Mishan, their superior light and temperature conditions and soil fertility (e.g., higher alkali-hydrolyzable nitrogen and available phosphorus) are key to forming thick stem bases (0.30 cm) and high biomass. However, the low root-shoot ratio (<0.56) suggests a risk of excessive above-ground growth, so attention should be paid to coordinating growth through water and fertilizer regulation and moderate water control, focusing on improving the root-shoot ratio. In unique tillering regions like Tailai, the advantage of early tillering is evident (tiller number 0.31), but the low strong seedling index (only 0.0399) reflects insufficient stem plumpness. It is necessary to utilize its tillering potential while enhancing stem strength by ensuring phosphorus and potassium supply and optimizing population light conditions. In compact stress-tolerant regions like Fangzheng and Fuyuan, environmental stresses such as spring low temperatures have shaped their morphology characterized by shorter plant height (<13.4 cm) but dense tissue. The extremely high plumpness (>23.7%) is their core stress resistance asset. This characteristic can be fully utilized, and growth can be moderately promoted through measures like heat preservation while ensuring robustness. The balanced robust seedling model in Qing’an and Suibin achieves synergy between high total dry weight (>4.57g) and reasonable structure (root-shoot ratio >0.73). It is a model of high harmony among environment, variety, and management, and can serve as a standardized reference example for achieving high-yield and robust seedling goals in similar suitable areas. This study provides clear data support and a decision-making basis for the regionalized application and optimization of this technique.

#### 3.4.3. Comprehensive analysis of site × year combinations.

The comprehensive results from multi-site and multi-year experiments showed that treatment (T1) had a significant enhancing effect on core seedling quality indicators compared to the CK control group. Among them, stem base width increased by 9.52% (P < 0.01); plant height increased by 6.82% and root length by 8.97% (both P < 0.05); and the strong seedling index increased by 12.41% (P < 0.01). Additionally, the tiller number showed a significant increase (0.18 per plant in T1 vs. 0 in CK), though the agronomic significance of this seedling-stage tillering requires further investigation. Treatment (T1) achieved comprehensive improvement in seedling quality, with positive regulatory effects on both morphological construction and physiological plumpness [25].The percentage increases in core seedling quality indicators of T1 relative to CK are shown in Fig 1.

**Fig 1:**
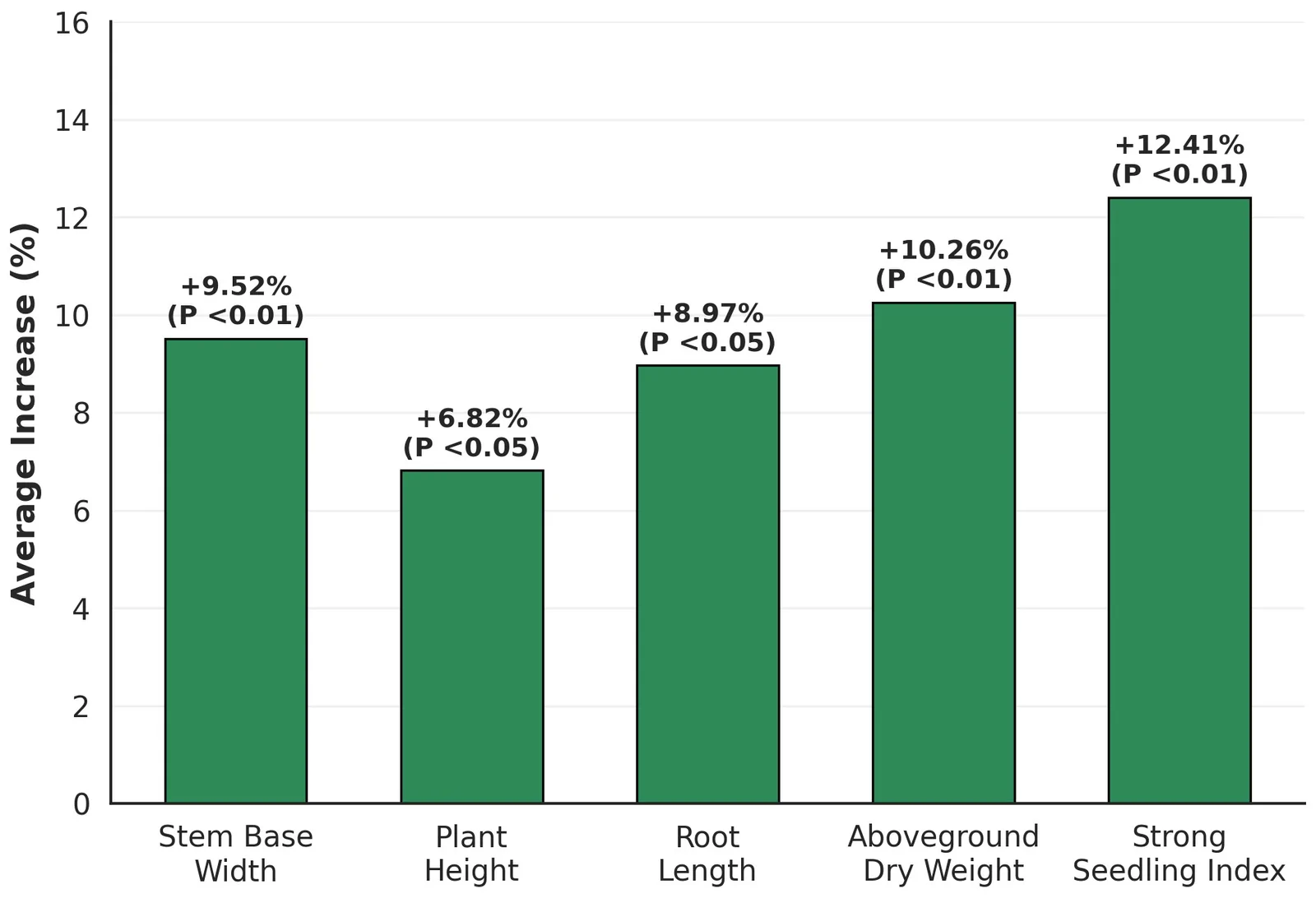
Percentage increase in core seedling quality indicators of the biodegradable film-based tray-free seedling system(T1) relative to conventional tray seedling raising (CK) across 21 site-year combinations. *P*-values from independent-samples *t*-tests are indicated above bars.

The heatmap of 21 site×year combinations (7 sites × 3 years) showed that the T1/CK ratios were all > 1.02, with no disadvantageous combinations, indicating good technical stability and wide adaptability. The ratios in Tonghe and Mishan were the highest across all years (indicated by dark green areas), with Mishan in 2024 having a strong seedling index ratio of 1.20, root length ratio of 1.18, and plant height ratio of 1.15. Fangzheng, Suibin, and Qing’an were moderately advantageous areas (light green to green), with ratios ranging from 1.08 to 1.12. Fuyuan had the lowest ratios (indicated by light yellow-green areas), but they still remained between 1.02 and 1.07, indicating that the technique can still exert a positive effect even under low-temperature and high-humidity environments. The ratios increased slightly over the three years at each experimental site. For example, the strong seedling index ratio in Tonghe increased from 1.13 in 2022 to 1.18 in 2024, confirming that the advantages become more prominent as the technique is refined [26].The heatmap of T1/CK ratios across 21 site-year combinations is presented in Fig 2, illustrating the regional and inter-annual stability of the technique.

**Fig 2:**
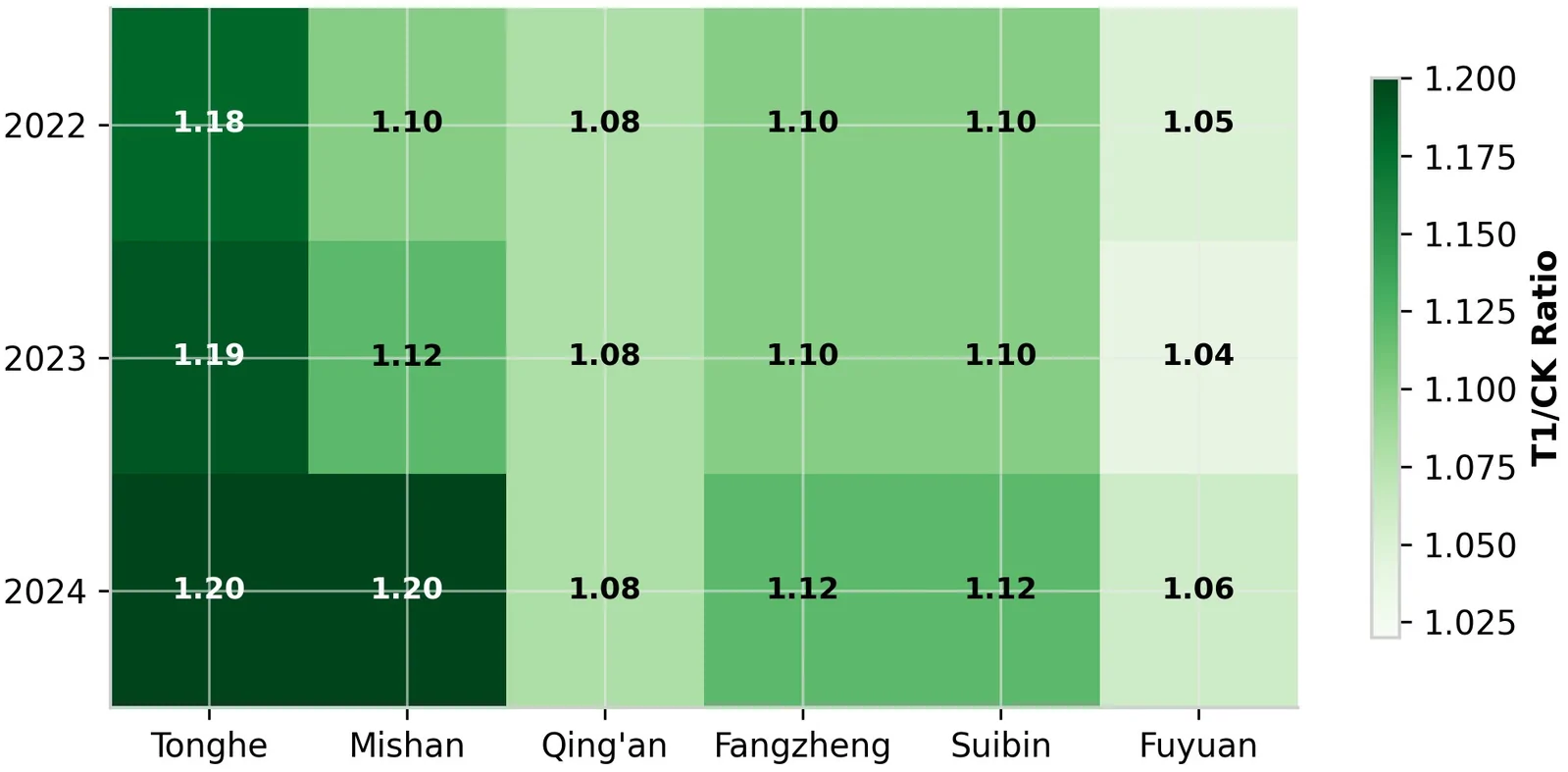
Heatmap of T1/CK ratios for core seedling quality indicators across 21 site-year combinations. Darker colors indicate greater relative advantages of T1 over CK. Data were standardized prior to visualization.

The significant genotype × environment × technique (G × E × T) interaction observed in this study underscores that seedling quality outcomes are not determined by technique alone. In high-biomass regions (Tonghe, Mishan), superior light-temperature conditions and soil fertility amplified the technique’s positive effects on stem base width and biomass accumulation. Conversely, in compact stress-tolerant regions (Fangzheng, Fuyuan), spring low temperatures constrained plant height but enhanced plumpness, suggesting that the technique interacts with environmental stress to shape distinct seedling phenotypes. Variety selection further modulated these interactions, as evidenced by the elevated strong seedling index of Lianhui 6730 at Suibin in 2024. These findings imply that regional promotion of this technique should integrate variety screening and local agronomic optimization rather than relying on standardized protocols.

## 4. Conclusions

1) This technique can significantly optimize the comprehensive quality of seedlings. Compared with traditional mechanical transplanting tray seedling raising, seedling height, root length, stem base width, dry weight per 100 seedlings, and the strong seedling index increased by an average of 6.82%, 8.97%, 9.52%, 10.26%, and 12.41%, respectively. It can stably maintain root-shoot balance and water metabolism, with no bacterial wilt or seedling blight observed throughout the experimental period, significantly enhancing the stress resistance potential of seedlings and laying a solid foundation for high and stable rice yields.
2) This technique simplifies the seedling raising process, reduces labor input by more than 30%, lowers the total cost of seedling raising by 15%–20% [15], and is compatible with mechanical sowing and transplanting.Replacing plastic trays with a biodegradable seedling film eliminates white pollution residues [27–29], aligning with the direction of green agriculture. Moreover, core indicators showed year-on-year improvement during the three consecutive years of trials from 2022 to 2024, demonstrating the stable and controllable effect of the technique.
3) This technique exhibits good regional adaptability and stability; the magnitude and pattern of treatment effects varied across ecological regions, indicating a significant technique-by-environment interaction. Data from 21 site×year combinations over three years showed that all treatment combinations (T1/CK ratio > 1.02) exhibited positive effects, validating the wide adaptability of the technique. Particularly, the increases in the strong seedling index, stem base width, and root length lay a solid seedling-stage foundation for rapid greening after transplanting, enhanced stress resistance, and the ultimate achievement of high and stable yields [30]. Future research should focus on long-term soil health monitoring following repeated application of biodegradable films, including assessments of soil microbial community dynamics, organic matter decomposition rates, and cumulative effects on rhizosphere ecology across multiple growing seasons.

## Supporting information

S1 Dataset Minimal dataset of seedling quality indicators for the biodegradable film-based tray-free seedling system (T1) and conventional tray seedling raising (CK) across seven experimental sites in Heilongjiang Province, China, from 2022 to 2024. The dataset includes morphological indicators (plant height, root length, stem base width, tiller number, leaf age), biomass indicators (fresh and dry weights, root-shoot ratios, dry-fresh ratios), and derived vigor indicators (strong seedling index, stockiness, plumpness). See Data Dictionary sheet for variable definitions and calculation formulas.
(XLSX)

S1 Fig Box-and-whisker plots of seedling morphological indicators across 21 site-year combinations. T1: Biodegradable film-based tray-free seedling system; CK: Conventional tray seedling raising. Boxes represent interquartile ranges (IQR), horizontal lines within boxes indicate medians, diamonds indicate means, whiskers extend to 1.5 × IQR, and red circles indicate outliers.
(JPG)

S2 Fig Box-and-whisker plots of seedling biomass indicators across 21 site-year combinations. FW: fresh weight; DW: dry weight. Other conventions as in S1 Fig.
(JPG)

S3 Fig Box-and-whisker plots of core seedling quality indicators across four ecological zones. Data demonstrate technique × environment interaction effects. SRI: strong seedling index; SBW: stem base width; RL: root length.
(JPG)
